# Developing 'robust performance benchmarks' for the next Australian Health Care Agreement: the need for a new framework

**DOI:** 10.1186/1743-8462-5-1

**Published:** 2008-04-25

**Authors:** Stephen J Duckett, Michael Ward

**Affiliations:** 1 Australian Centre for Economic Research on Health, University of Queensland, Brisbane, Australia; 2 University of Queensland, Brisbane, Australia

## Abstract

If the outcomes of the recent COAG meeting are implemented, Australia will have a new set of benchmarks for its health system within a few months. This is a non-trivial task. Choice of benchmarks will, explicitly or implicitly, reflect a framework about how the health system works, what is important or to be valued and how the benchmarks are to be used. In this article we argue that the health system is dynamic and so benchmarks need to measure flows and interfaces rather than simply cross-sectional or static performance. We also argue that benchmarks need to be developed taking into account three perspectives: patient, clinician and funder. Each of these perspectives is critical and good performance from one perspective or on one dimension doesn't imply good performance on either (or both) of the others.

The three perspectives (we term the dimensions patient assessed value, performance on clinical interventions and efficiency) can each be decomposed into a number of elements. For example, patient assessed value is influenced by timeliness, cost to the patient, the extent to which their expectations are met, the way they are treated and the extent to which there is continuity of care.

We also argue that the way information is presented is important: cross sectional, dated measures provide much less information and are much less useful than approaches based on statistical process control. The latter also focuses attention on improvement and trends, encouraging action rather than simply blame of poorer performers.

## Background

The Council of Australian Governments (COAG) communiqué issued after its 20 December 2007 meeting provided a serious challenge for the yet to be established National Health and Hospitals Reform Commission (NHHRC) in specifying terms of reference which included a requirement that:

by April 2008, the Commission will provide advice on the framework for the next Australian Health Care Agreements (AHCAs), including robust performance benchmarks in areas such as (but not restricted to) elective surgery, aged and transition care, and quality of health care.

At the same meeting COAG established a Working Group on Health and Ageing as a Commonwealth-state officer level group that to some extent parallels both the role and timelines of the Commission. The Working Group's indicative forward work plan for 2008, as endorsed by COAG, includes:

Consideration of a Commonwealth/State agreement across the full range of health and wellbeing issues, including outcomes, measures of progress and accountability arrangements.

Developing performance measures for the health sector is complex; getting them agreed between Commonwealth and states when a considerable amount of funding is at risk will be an even greater challenge. Developing a performance measurement or benchmarking framework includes a number of steps. The first step for the NHHRC is a framework for the AHCAs. Performance measures within that framework then need to be developed and agreed.

## Frameworks for accountability

There are a number of choices about frameworks for the health system. As Simon [1] and Lindblom [2] have pointed out, humans usually exhibit "bounded rationality" when making decisions or planning, where they cannot consider all aspects of a problem and so limit their consideration to those aspects which are familiar. This is a hazard wherever the subdivisions of specialisation are needed to manage an increasingly complex production environment, well exemplified by healthcare. Compartmentalisation and the "mind maps" of a system shape the choices decision makers are prepared to consider and thus the relative emphasis of policy initiatives.

The first task of developing a framework and consequential performance measures is thus to determine what our mind map of the health system will be, that is how to define and describe the health care system. Conventional approaches to this task are typically influenced by the history and constraints of analytical approaches and by the current characteristics of the system. In the former case Weaver [3] for instance identified three approaches: classical two variable problems at the least complex end of the problem spectrum and statistical mechanics at the other where thousands of variables interact in disorganised complexity, with a mid zone of organised complexity where a medium numbers of variables interact. Unfortunately it is in this mid zone, the least well understood or analytically developed, that most important healthcare problems reside and where new approaches are needed. In the latter case, we most commonly describe the system in institutional terms such as primary care, secondary or acute care and long term care. This institutional approach was challenged by COAG decisions under the Hawke/Keating Government, which attempted to shift the frame used in health system design from an institutional/provider oriented one to one more focussed on "meeting people's needs" [4]. In contrast to the institutional approach, the COAG approach identified three different sorts of needs: primary/episodic needs, chronic or 'coordinated care' needs, and acute care needs, all of which were situated within a population health framework.

The critical difference between the institutional and the population approaches is the identification of needs for care co-ordination, with the then Labor Government's response being to sponsor the coordinated care trials [5]. This frame has subsequently fallen into disuse as the institutional frame of primary or secondary care regained ascendancy. Insofar as the Howard Government responded to these chronic disease or coordinated care needs, policies were developed within an institutional framework; the main Howard Government response to this different class of needs was the Enhanced Primary Care package. Enhanced Primary Care, although an advance on the pre-existing primary care episodic funding model, did little to establish new approaches to deal with the very different needs that were beginning to be recognised with the ageing of the population and changing health status. If the population focus had continued, Australia's health care system could now be very differently positioned to respond to the growth in chronic disease. The argument here is not to adopt the old COAG framework of the early to mid 90s, but rather to illustrate that the choices about how we describe the health system can influence the emphasis of policy. Those choices about the framework of the system are thus going to be critical to the new NHHRC and the officer-level working group.

A second choice that is implicitly made in decisions about descriptions and measures of the health care system is whether to emphasise cross-sectional descriptions and static performance of systems, such as the number of patients treated or beds open, or de-emphasise these and emphasise measures of the dynamics of the system. Static measures predominate, as if the body were to be described only by its anatomy and not its physiology. A systems dynamic approach emphasises the stocks and flows of a system and the reality that the stocks are affected by the flows and both have to be modelled, designed and measured [6]. This approach is critical in the health system with its intricate interdependencies but unfortunately it has been shown that high intelligence and educational status are no guarantee that even simple stock and flow problems can be solved or even understood [7]. What is important to take from this then is that any systems of performance measures needs to focus not only on the institutions or single components of services but on the interactions between them.

Benchmarking (and performance measurement generally) is about evaluation and 'valuing'. This requires a third choice about what 'value' attributes will be highlighted in the design of the performance system. This is as critical as the choice of the health system framework.

**Figure 1:**
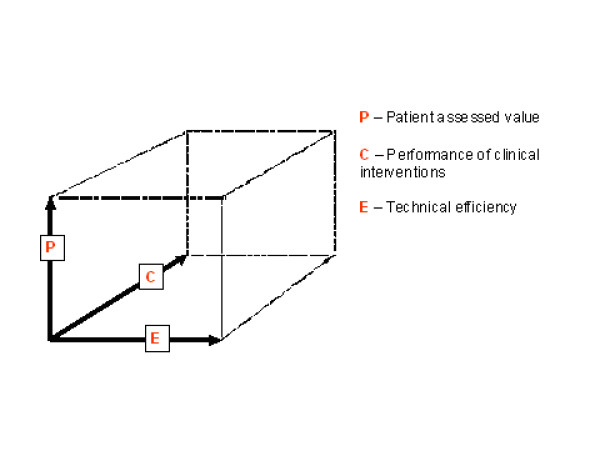
The health care evaluation 'value cube'.

The health care system is characterised by multiple products (acute care, research, teaching) with the 'value' of each of those products being characterised along multiple dimensions. In the case of acute care, for example, value can be measured from the perspective of each of the three players: the patient, clinicians, and the funder (financial or technical efficiency perspective). These perspectives may overlap but often do not, and are sometimes in open conflict [8,9]. Thus a healthcare facility may perform well from any one of these perspectives or dimensions and poorly on either or both of the other two. The 'value' of the a service should therefore be conceptualised as being distributed across a "value cube" rather than as a one-dimensional continuum or a Cartesian plane (see figure 1)

Each dimension of this cube incorporates in turn a number of distinct elements and thus might itself be regarded as multi-dimensional. This provides the framework with the additional attraction of scalability: the three dimensions may be scaled to examine an individual subcomponent or to view the progression from the characteristics of an individual patient, through aggregation at clinical service or hospital level up to whole populations.

A patient's journey through the health care system provides a useful framework for identifying some of the elements that contribute to patient assessed value and which might be the subject of performance benchmarks. Kenagy et al, in a article describing the journey of one of its authors, use the term 'service quality' for this perspective on value [9]. Critical elements of patient assessed value might include:

• A patient's journey involves a number of separate providers. The quality of this journey is measured in large part by how effectively the transitions are managed from a patient perspective i.e. to what extent is there *continuity of care *in the transitions including the transition from primary care to acute care (and back again), from acute care to any necessary long stay care and so on. The focus should be on the patient's experience e.g. to what extent is the patient asked to repeat information at different stages of the journey? To what extent are diagnostic tests repeated by different providers? To what extent does a patient stay in an acute hospital facility inappropriately prior to relocating to a residential aged care facility?

• Patient perceptions are also affected by the *timeliness *of access and this is typically measured in terms of waiting times. The policy focus has been on waiting times for elective surgery, but waiting times for the whole journey are important. In Sweden for example a benchmark described as 0-7-90-90 has been promulgated, based on a zero wait for access to a call centre, no longer than a 7 day wait to be seen for a primary care consultation, no longer than a 90 day wait for a secondary care consultation and no longer than 90 day wait for treatment to be commenced, including any necessary hospital admission [10,11]. If this benchmark were adopted in Australia, the secondary care wait time would apply to both consultations in a specialist's private rooms as well as in hospital outpatient departments. The English National Health Service has adopted a more ambitious, integrated target: 18 weeks from first referral to treatment [12].

• Patients commence their journey because of the need for symptom relief and/or functional improvement and thus a critical component in assessing value from a patient's perspective is the extent to which the *patient's expectations *of improvement have been met. In Australia we rarely systematically measure patient's expectations or whether they are realised. Significant work has been undertaken in the United Kingdom in terms of 'patient reported outcome measures' [13]. A significant measurement task would be required to develop and implement appropriate measures if Australian benchmarks about responding to patient symptomatic and functional improvement expectations were to be adopted. We would need to reach an agreement on the measures, introduce them and develop reasonable standards for what can be achieved in terms of improvement of symptom relief and functional improvement. Consideration would need to be given as to whether generic measures (which would apply across all specialties) or condition-specific measures are better for this purpose.

Patients expect health care to improve their condition, not worsen it. Low mortality, morbidity and risk of adverse events are obviously critical aspects of patient expectations and could thus be included in this dimension. But measurement and identification of whether a facility is truly different on these indicators is complex and still a developing science (see discussion below).

• A third facet relates to *the patient experience*, in particular, the way a patient is treated during the course of their care, what Murray and Frenk refer to as 'respect for persons' [14]. This would include measures such as being treated with dignity, respect for autonomy and confidentiality, adequacy of the information provided to them (on admission, during the course of treatment, at discharge), and so on.

• Finally a fifth measure is the *cost *to the patient (costs to society are the focus of the third dimension of the value cube). To what extent were there out of pocket costs involved in accessing treatment? These costs include not only payments to health service providers but also any accessibility costs such as costs of travelling and accommodation to obtain patient care.

Zeithaml et al have developed a comprehensive list of generic dimensions of a 'customer' view of service quality that could be used to flesh out other components of patient assessed value [15] but the measures proposed here include measures of flow or co-ordination (first two dot points above), outcome (third dot point) and processes of care (last two points).

Clinician defined indicators are usually condition-specific, so those markers for representative conditions should be included preferably, and perhaps only, if they define evidence based best practice interventions for that condition (e.g. whether appropriate diagnostic tests have been performed and/or whether appropriate drugs have been prescribed). As a more stringent test the proportion of *all *proven evidence based best practice interventions for the relevant condition that have been implemented should ideally be included [16].

Given the link between clinical teamwork and outcomes [17,18], another process measure of clinical quality might be a measure of teamwork and communication. The increasing importance of chronic disease suggests that benchmarks should also be developed to focus on system performance in this area, including the extent to which the health system/facility/provider facilitates patient self-management. Here dynamic measures might be most important: higher rates of admissions to hospitals for avoidable conditions might be regarded as system failures and poor clinical quality in primary care.

Outcome measures of clinical quality, such as complications, readmission or mortality rates, could also be considered as part of a measurement and benchmarking regime. However, the relative place of outcome versus process measures of clinical quality is subject to some debate [19-22]. Process measures have the advantage of being clearly and unambiguously the responsibility of the clinician(s) and there is a clear action path from deficits in process measures to process improvement. Good performance on process markers seems to be associated with even better than expected performance on outcome measures, suggesting process measures serve as markers for other, unmeasured aspects of care [23]. There is certainly ample scope for improvement in terms of processes as, if Australian experience replicates that of the United States in terms clinical practice, on average only about half (range 10–80%) of those with a valid indication receive appropriate evidence based therapies or interventions [24].

Use of outcome measures is more complex from both a statistical and management perspective. Outcome measures require greater levels of statistical sophistication to perform appropriate risk adjustment and identify how much of any variation is due to chance. Brennan et al put the argument strongly:

This notion (of individual accidental death) oversimplifies the causal realities of iatrogenic injuries, overpromises on achievable gains, and threatens to skew priorities in quality-improvement initiatives. Moving away from a focus on saving lives solely by preventing errors and instead emphasizing the implementation of evidence-based practices to improve the quality of care more generally will yield better long-term results [19].

Porter and Teisberg have argued for value to be restored to healthcare through competition based on outcomes rather than processes of care [25], this is understandable but as discussed above process markers are more practicable and, if evidence based, process improvement should lead to gains in outcomes. In a sense, outcome measures can only flag a difference for further investigation: to what extent is any difference identified due to coding error, unadjusted case mix or indeed process variation [26]? Simply publishing that hospital A has a worse outcome than hospital B does nothing to improve quality of care. What is important is that hospital A investigates the reason for variation and addresses any causes within its control [27]. This might then transform an outcome measure to a process one: to what extent is any identified variation addressed in a timely way?

The final dimension of value is one relating to efficiency. From an economic perspective, efficiency has three key elements: *technical efficiency *which broadly speaking can be defined as efficiency in production (operationalised normally as inputs divided by outputs e.g. cost per patient treated) and is represented on the third dimension of the value cube, *allocative efficiency *(which may be defined as ensuring that there is an optimum allocation of resources in the sense that the marginal dollar spent on any program yields the same level of marginal benefit as the last dollar spent in any other program and thus involves a focus on outcomes, such as quality adjusted life years gained relative to inputs) and *dynamic efficiency*.

Technical efficiency is the easiest to measure of the three elements and, with total expenditure (which is not a critical element from an economic perspective as what is relevant is whether there are returns from expenditure, not the absolute amount spent), tends to be the main foci of 'economic' considerations. Importantly, technical efficiency and clinical performance, although arrayed on different dimensions, are not inherently antithetical as high clinical quality can be associated with lower, more appropriate, use of inputs by, for example, eliminating duplicate diagnostic tests [28].

A simple decomposition shows that allocative efficiency incorporates technical efficiency and moves beyond it to incorporate a focus on effectiveness, being the outcomes achieved for each unit of output:

**INPUT/OUTCOME **(Allocative efficiency) = **INPUT/OUTPUT **(Technical efficiency)* **OUTPUT/OUTCOME **(Effectiveness measured as inverse)

Allocative efficiency creates a link with both patient assessed value and clinical quality, as outcomes can be measured from either perspective (or both). Allocative efficiency is thus achieved when both quality and technical efficiency are optimised (the best outcome for each output, patient treated).

An economic perspective also involves a focus on *dynamic efficiency*, which looks to development and innovation of new products and processes to improve technical and allocative efficiency over time. One measure of this might be investment in, or outputs from, clinical and health services research and development. So, just as the patient and clinical perspectives of value incorporate a number of distinct elements, an economic perspective incorporates different aspects of efficiency, all of which should be measured.

These final two elements of efficiency are integrative in that achievement of allocative or dynamic efficiency requires performance on multiple axes. In a sense a focus on allocative and/or dynamic efficiency entails wider societal perspectives, such as the perspective of the taxpayer or 'the community'. It also provides a link to consideration of population health issues (discussed below) in the context of the tradeoffs between investments in curative versus preventive interventions.

The value cube does not incorporate a separate dimension for equity even though measures of both patient assessed value and clinician quality of practise could reveal different results in different populations. An acute care facility, for example, could be excellent in terms of patient assessed value for the majority of its patients, but poor for distinct sub groups of the population characterised in terms of race, gender, geography and so on. Similarly, clinical outcomes of care for different sub groups of the population may be different. Equity then is not treated in this model as a separate dimension of the main axes but rather needs to be considered as part of the distributional properties of each of the two relevant axes.

The fourth issue that needs to be addressed are the attributes of the indicators themselves. The management literature sums up desirable attributes of an indicator with the acronym SMART. Although the acronym is widely used, there is some disagreement as to what the constituent initials stand for. There is agreement that indicators need to be Specific and Measurable. The A usually stands for Achievable, (sometimes the lexically similar, Attainable). The A may also refer to Agreed, Appropriate, Action-oriented. Young et al have added Aware to this list [29]. The R can stand for Relevant but may also be Realistic (if A is not Achievable), Rewarding or Results-oriented. T is Timely (sometimes Time-bound/Time-based or Tactical). Unfortunately all are desirable properties of indicators, but rarely are they adequately considered in design of indicators and benchmarks.

### The importance of statistical process control

Whatever measurements are selected in whatever dimension, it is essential that the most appropriate and effective methods are used to monitor trends and to demonstrate any significant positive and negative changes. Such changes need to be followed at all levels, individual patient, clinical service, facility or population. As discussed above biomedical science has been driven by one legacy of statistical mechanics, the randomised double blind controlled trial. This is understandable given the explanatory power of such trials, but in the messy world of clinical practice they may be of little help – patients must be treated that would probably have been excluded in the trial and answers are needed in days or weeks rather than years.

Fortunately a more appropriate methodology, statistical process control, is available although relatively neglected in healthcare until recent times [30]. This may reflect its humble manufacturing industry origins, but there is increasing recognition of the value of these techniques in a wide range of health care activities including the monitoring of individual and service safety and quality of care [24,31-33], acquisition of surgical and procedural skills [34,35] and patient flow [36]. The particular value of statistical process control is the rapid access it provides to significant trends in local information without the need for carefully matched control groups. In this context, Priesmeyer has developed a method of phase plane analysis for demonstrating changes in two related time series functions that seem useful for tracking both macro-organisational performance measurements [37] and micro-clinical aspects such as the expected recovery trajectory after surgery [38]. We believe that this approach can be readily and usefully extended to the three dimensions as the value cube.

## Measuring the Performance of Population Health Activities

The preceding discussion has focused on measuring the performance of services, but the care system sits within and is impacted by a wider environment. The health system (as distinct from the care system) also incorporates population health activities, benchmarking these activities involves somewhat different considerations. In broad terms the goal of population health programs is to protect and improve the health status of populations. Achievement of this goal involves providing direct services to individuals (e.g. screening services) and provision of programs with no identifiable individual clients (e.g. food hygiene regulation).

Population health programs also assume a monitoring or 'tracking' role with respect to the overall health status of the population. This 'tracking' role usually incorporates measures of health outcome (morbidity or mortality rates, incidence and prevalence rates). It is not proposed here to incorporate them as measures of accountability of the *health system*, partly because trends in mortality or morbidity generally cannot be influenced by decision makers within reasonable time frames and partly because outcome measures like mortality and morbidity are significantly influenced by factors exogenous to the health system (in this context, probably more accurately called the health services system). This is not to say that tracking health outcomes is not useful over the longer term or, indeed, as part of an evaluation of highly targeted interventions such as a breast screening program.

Those aspects of population health programs which involve direct service provision should be evaluated along the lines of the service dimensions outlined above: the same issues of patient, clinical and economic perspectives are just as relevant to population health delivery as they are to community health or hospital services.

Other than using input or instrumental measures, performance measures for programs where there are no individually identifiable clients are somewhat more difficult to develop. However, output or outcome measures can be derived from measures of process success or process failure using hospital or other service databases, such as admission rates for ambulatory care sensitive conditions [39-41].

## Conclusion

Health services and health systems are complex, generally involving provision of multiple products. There are also multiple attributes of the service system that are relevant to assessing and determining the 'value' of the system. Together, these characteristics mean that developing a framework for accountability is exceedingly complex.

Developing a framework for the health care system has to avoid the same pitfalls involved in specifying objectives so eloquently described by Nienaber and Wildavsky:

Of objectives it can be said that they invariably may be distinguished by three outstanding qualities: they are multiple, conflicting and vague.... The assumption that objectives are known, clear, and consistent is at variance with all experience. Objectives are not just out there, like ripe fruit waiting to be plucked; they are man-made (sic), artificial, imposed on a recalcitrant world. Inevitably, they do violence to reality by emphasising certain activities (and hence organisational elements) over others. Thus the very process of defining objectives may be considered a hostile act. It they are too vague, no evaluation can be done. If they are too specific, they never encompass all the indefinable qualities that their adherents insist they have. If they are too broad, any activity may be said to contribute to them. If they are too narrow, they may favour one segment of the organisation against another [[42], p10].

So the choice of the framework, measures and associated benchmarks in the new Australian Health Care Agreements is not a technocratic or value neutral process. It involves choices on what will be measured, prioritising some aspects of 'value' over others. In the context of a system with responsibilities for different parts of the system resting with different levels of government, deciding which areas of the system will be subject to benchmarking is a quintessentially political act: what benchmarks will relate to performance of states and territories and what (if any) benchmarks will be developed for processes for which the Commonwealth is accountable?

But the problem will not be solved by allowing every interest to have its indicator. Too many COAG indicators will effectively vitiate any accountability process by overwhelming reviewers with too many data elements. On the other hand, as Nienaber and Wildavsky point out, too few indicators will inevitably mean that some important areas for accountability will be neglected. Consolidating multiple indicators does not solve this issue as assigning weights associated with each component of the composite indicator involve the same value or political choices, with the added risk of loss of transparency [43]. Developing the right balance of indicators is thus difficult. Balance will only be achieved however if the particular indicators are chosen to reflect the particular experiences and aspirations of the key players: the patient, the provider or clinician and the purchaser or funder of services. At the moment financial/efficiency indicators are most widely available and precise clinical practice indicators are usually more widely available but often less precise, and patient indicators hardly available at all, often poorly specified, and rarely linked to the other two dimensions.

This balance also needs to be evaluated and displayed using a framework that makes transparent not only the interrelationships among these diverse perspectives but the inevitable compromises that are part of any optimised health care delivery system. We suggest that the 'value cube' framework has such potential and the capacity to graphically represent the global return on investment of health care funding in a scalable form. In essence this approach can concisely define and demonstrate value in healthcare by showing whether clinical intervention(s) produce the desired patient outcome(s) efficiently and effectively. This need for this type of analysis will assume increasing importance with the increasing implementation of 'pay for performance' funding models [44-49].

So where do we go? The way forward may require more time than allowed the NHHRC and the Working Group as there are two tasks which both need to be completed well: one to develop a framework and appropriate, technically-sound measures and the other to build acceptance of the approach. Unfortunately these are not separate and distinct tasks as participation in design may be necessary for acceptance. The goal here is widely accepted technically sound benchmarks, within a short time frame. A formidable task! The compromise may be to develop an interim list of benchmarks in the short term with a clear agenda, process, time frame and funding to develop better measures, benchmarks and reporting approaches over a more realistic time frame.

## Authors' contributions

MW developed the original idea of the value cube which has been refined in the writing of this paper. Both authors contributed to writing and approved the final manuscript.
